# Ecotoxicological Characterization of Lithium as a “Timebomb” in Aquatic Systems: Tadpoles of the South American Toad *Rhinella arenarum* (Hensel, 1867) as Model Organisms

**DOI:** 10.3390/toxics12030176

**Published:** 2024-02-25

**Authors:** Paola M. Peltzer, Ana P. Cuzziol Boccioni, Andrés M. Attademo, María F. Simoniello, Germán Lener, Rafael C. Lajmanovich

**Affiliations:** 1 Laboratorio de Ecotoxicología, Facultad de Bioquímica y Ciencias Biológicas, Universidad Nacional del Litoral, Santa Fe S3001XAI, Argentina; paolapeltzer@hotmail.com (P.M.P.); mattademo@hotmail.com (A.M.A.); 2 Consejo Nacional de Investigaciones Científicas Técnicas (CONICET), Buenos Aires C1425FQD, Argentina; germanlener@gmail.com; 3 Cátedra de Toxicología, Farmacología y Bioquímica Legal, Facultad de Bioquímica y Ciencias Biológicas, Universidad Nacional del Litoral, Ciudad Universitaria, Santa Fe S3001XAI, Argentina; fersimoniello@yahoo.com.ar; 4 Instituto de Investigaciones en Físico-Química de Córdoba-CONICET, Departamento de Química Teórica y Computacional, Facultad de Ciencias Químicas, Universidad Nacional de Córdoba, Córdoba X5000GYA, Argentina

**Keywords:** Li, environmental risk, tadpoles, aquatic contamination

## Abstract

The aim of this study was to evaluate the acute lethality and chronic sublethal effects of lithium (Li) on *Rhinella arenarum* tadpoles as model organisms. First a 96 h toxicity assay was performed by exposing tadpoles to Li concentrations from 44.08 to 412.5 mg L^−1^ to estimate the mortality, and lethal and sublethal effects. Another bioassay was carried out by exposing tadpoles to two environmentally relevant Li concentrations (2.5 and 20 mg L^−1^) for one and two weeks. The sublethal effects of Li on tadpoles were evaluated by analyzing biochemical, genotoxic, and physiological biomarkers. The mortality in Li-exposed tadpoles increased over time. The median lethal concentration (LC_50_) ranged from 319.52 (281.21–363.05) mg L^−1^ at 48 h to 66.92 (52.76–84.89) mg L^−1^ at 96 h. Exposure to Li at 2.5 and 20 mg L^−1^ induced alterations in enzymes related to detoxification, antioxidant, and hepatic mechanisms, endocrine disruption of thyroid hormones, genotoxicity, and effects on the physiology of the heart and gastrointestinal systems. Tadpoles exposed to the highest concentration in the chronic bioassay (20 mg L^−1^ Li), which is the concentration commonly recorded in Li mining sites, showed significant mortality after one week of exposure. These results warn about the high ecotoxicological risk of Li as a contaminant of emerging concern for amphibians.

## 1. Introduction

Lithium (Li) is a silver-white-colored soft metal belonging to the alkali metal group, and is found in brines (saline waters), hard rock pegmatite ore, and clay minerals. Brines are the main Li sources recorded in different stages of geological development in North and South American countries such as Argentina, Bolivia, Chile, and the United States [1]. However, during the last two decades, the uses of Li have drastically increased over 256% due to the growing intensive demands for this fuel by the green energy revolution and technologies [2,3]. Currently, Li is used in different industries, including heat-resistant glass and ceramics, Li grease lubricants and greases, and household appliances. In addition, in the next decade, increases in the demand for Li-ion batteries for electric-powered vehicles and mobile devices will be a strong driver of Li consumption, with the global demand expected to reach 2.4 million metric tons of Li carbonate equivalent [4,5]. In South America, Li is extracted mainly from continental brines that are geographically restricted to the Li Triangle (northwest Argentina, southwest Bolivia, and northern Chile), with an estimated 50–85% of Li-rich continental brine deposits worldwide [1,6]. The method used to extract Li from continental brine deposits is open air evaporation to concentrate the brine. This method implies high levels of pollution (most strongly associated with open pit mining) and unsustainable use of water, both of which alter the environmental quality and threaten the ancestral lands of indigenous communities [7,8].

Studies on Li concentrations in surface waters and on the toxic effects of Li on aquatic organisms are scarce [9]. Environmental concentrations of Li vary between 0.014 and 14 mg L^−1^ [10], whereas those in salt flat regions (such as the “Puna de Atacama” in Bolivia) vary between 0.2 and 20 mg L^−1^, and can increase up to 400 mg L^−1^ [11]. Based on United States Geological Survey (USGS) data collected from 17 countries, the global geographic distribution of Li in water bodies could be classified in a wide range: <0.06, 0.07–3, 3.1–10, 10.1–20, and >20 mg L^−1^ [3].

Although several data sets are available on Li accumulation in the food chain, few studies have determined the effects of chronic exposure to Li through water intake on human health and biodiversity [3,12]. In particular, many wildlife species in the areas of Li extraction are wetland-dependent [13]. These include Andean flamingos (*Phoenicopa-rrus andinus* and *P. jamesi*), which breed specifically in the region and feed on brine shrimp in Andean hypersaline lakes [14], and endemic amphibian species of the threatened genus *Telmatobius* [15]. Regarding the former, Gutierrez et al. [16] pointed out that the size of flamingo populations is negatively correlated with Li mining. Several studies have also reported negative impacts of Li extraction in South America, where brine pumping has caused groundwater levels to drop [17] and altered the quality of wetland ecosystems and the species dependent upon them such as fishes and amphibians [15]. In this context, a few studies have described the effects of Li on fish and amphibian species as related to detrimental effects on embryonic development and organogenesis that lead to severe teratogenesis or abnormal development and growth [18,19,20,21].

On the other hand, Li and its compounds are also used for human medicine, being prescribed as a psycho-pharmaceutical against bipolar disorder since its discovery in 1970 [22]. Studies on the toxicity of Li have determined that Li could have a mechanism analogous to that of copper, which interferes with energy production and ion regulation [23]. Some of the direct targets of Li are Mg-dependent intracellular enzymes [24]. In this regard, Srinivasan et al. [25] provided evidence of competition between [Li^+^] and [Mg^2+^] for binding sites on guanine-nucleotide-binding proteins (G proteins). Moreover, chronic Li exposure has been found to have effects on the modulation of the synaptic function in nerve terminals [26]. In this context, El-Tekreti and Çilingir Yeltekin [27] demonstrated that Li has a toxic effect on brain metabolism by inhibiting antioxidant activities. It has also been demonstrated that Li induces changes in the sodium channels that decrease intracellular potassium, which increases the voltage of cardiac myocytes, causing electrical instability in the atria and ventricles [28]. Moreover, exposure to Li causes cytotoxic and genotoxic effects related to damage to the antioxidant/oxidant balance in cells [29] and induces thyroid abnormalities [30].

Studies of multiple biomarkers in amphibians have found that these biomarkers can be inferred as early warning signs of effects on the ecological health of an environment [31,32]. The lack of specificity of a single biomarker has led to an increase in the use of different biomarkers in ecotoxicological risk evaluation and the characterization of a contaminant [33]. In amphibian tadpoles, the ecotoxic responses can be examined by different hierarchical levels of biomarkers such as biochemical, cellular, tissue-, organ-, system-, and whole-organism-level markers, making an ecological study relevant [32].

Recently, water pollution has been pointed out as a “timebomb” (*sensu* Tozer et al. [34]) that threatens aquatic ecosystems in South America, which highlights the urgent need for its control, mitigation, and remediation by governments. In this context, the aim of this study was to evaluate the acute lethality and chronic sublethal effects of Li as a contaminant of emerging concern on tadpoles of *Rhinella arenarum* (Hensel, 1867). A first 96 h toxicity assay was performed by exposing tadpoles to a wide range of Li concentrations in order to estimate mortality, and lethal and sublethal Li concentrations. Another chronic bioassay was carried out by exposing tadpoles to two environmentally relevant Li concentrations (2.5 and 20 mg L^−1^). Biochemical, genotoxic, and physiological biomarkers were analyzed for the characterization of the sublethal effects.

Under the current scenario of the global demand for Li extraction from brines, ecotoxicological risk evaluations of Li are urgently needed to ensure that green technologies do not lead to a decline in wetland species such as amphibians.

## 2. Materials and Methods

### 2.1. Chemicals

In nature, Li does not occur in its free form as a reactive element [35]. It can be found associated with several species and mineral components, forming oxo-salts such as Li carbonate (Li_2_CO_3_), clays such as spodumene, and Li chloride (LiCl), an ionic salt [8,36,37]. In crystallochemical terms, Li is accommodated as a Lispecies in all those structures. The common method used in the industrial brine exploitation of Li is the evaporitic process, mainly due to its low-cost technology. This process involves successive stages of brine concentration by solar and wind evaporation in large ponds [6]. After the concentration process, several waste salts are produced in the purification steps to finally obtain LiCl and then Li_2_CO_3_ [7]. Each step involves large volumes of fresh water and inevitably produces LiCl as a pollutant [7,13]. In the present study, we used LiCl 99% (CAS N° 7447-41-8, Anedra^®^ Research, Buenos Aires, Argentina) as the Li source as it has been used in ecotoxicological assays on amphibian tadpoles as model organisms [19,21,38]. For simplicity, in the rest of the text, we refer to it as Li. Li treatments were prepared with dechlorinated tap water (DTW) with the following chemical properties: pH 8.1 ± 0.05, conductivity 410 µmhso/cm^−1^, dissolved oxygen concentration 5.5 ± 1.5 mg L^−1^, and hardness 83 mg L^−1^ as CO_3_Ca at 24 ± 2 °C.

### 2.2. Study Species

*Rhinella arenarum* (Bufonidae) is a South American toad species with a wide geographic distribution, including Brazil, Uruguay, Paraguay, Bolivia, and Argentina [39]. In Argentina, it is found in almost all provinces from Chubut northward [40]. It has been widely used as a model animal in laboratory studies for physiological, anatomical, and toxicological analyses [41,42,43]. Moreover, this species is of particular interest for the present study because it is one of the most abundant amphibian species in the Li Triangle area in Argentina.

Tadpoles (n ≈ 1000) of *R. arenarum* were collected from small temporary ponds located in the floodplain of the Paraná River (31°11′31″ S, 60°9′29″ W), which is considered natural and contamination-free according to previous studies [44,45]. The collection was allowed by the Ministerio de Ambiente of the Province of Santa Fe (EXP. N° 02101-0026248-0), Argentina. Tadpoles were immediately transported in DTW to the laboratory and acclimated to a 12 h light/dark cycle and 24 ± 2 °C before the assay. The experiments were performed with tadpoles of the same Gosner Stage (GS) [46,47] and size, which were selected from the collected ones.

### 2.3. Experimental Design

#### 2.3.1. Acute Lethality Test

An explorative acute 96 h toxicity assay was performed with tadpoles of *R. arenarum* (GS 26) exposed to a wide range of Li concentrations (44.08, 56.51, 72.45, 92.89, 119.09, 152.68, 195.75, 250.5, 321.75, and 412.5 mg L^−1^) in triplicate. A negative control treatment (CO) with DTW was also added in triplicate. Each replicate consisted of ten tadpoles in 1 L of test solution contained in a glass flask. The bioassay was maintained under fixed lab conditions (24 °C ± 2 °C, 12 h light/dark cycle). Tadpole mortality was recorded and dead animals were removed every 24 h. Cumulative mortality and survival rates for each treatment, as well as the median lethal concentration (LC_50_) and the no- and lowest-observed-effect concentrations (NOEC and LOEC, respectively) were calculated every 24 h, up to 96 h of exposure.

#### 2.3.2. Chronic Bioassay

Another bioassay was carried out for the analysis of different sublethal biomarkers. Tadpoles were exposed to three treatments: (A) a negative control with DTW (CO); (B) 2.5 mg L^−1^ Li (Li 2.5), which is a concentration that has been considered to be environmentally safe in South America [21,38]; and (C) 20 mg L^−1^ of Li (Li 20), which is the maximum Li concentration reported for Argentinean water bodies [3]. Chronic exposure to all treatments was originally planned to be for two weeks, an experimentally standardized time before tadpoles reach the prometamorphic phase of development (GS 36–41). However, in the Li 20 treatment, tadpole mortality exceeded 10% after the first week. Consequently, two exposure times were then evaluated: one week for the three treatments (CO, Li 2.5, and Li 20), and two weeks only for the CO and Li 2.5 treatments. The treatments were performed in triplicate, with n = 10 tadpoles in a 1 L glass flask for each replicate. The solutions were renewed every 48 h. Tadpoles were fed equal rations of boiled lettuce, which were added to each tank every time the solutions were renewed. The laboratory conditions were the same as those of the acute test and remained constant during the entire bioassay.

### 2.4. Biomarkers

#### 2.4.1. Biochemical Biomarkers

To determine the activities of different enzymes (glutathione-S-transferase, GST; carboxylesterase, CbE; alkaline phosphatase, ALP; alanine aminotransferase, ALT; and aspartate aminotransferase, AST) and thyroid hormone (T4) levels at the end of each treatment exposure, n = 8 tadpoles per treatment were weighed (g) and homogenized (1:10, *w*/*v*) in ice-cold 25 mM sucrose, 20 mM Tris-HCl buffer (pH = 7.4) with 1 mM EDTA, using a polytron tissue grinder. The homogenates were then centrifuged at 10,000 rpm at 4 ± 1 °C for 15 min, and stored at −80 °C until the analysis of biochemical biomarkers.

##### Enzyme Activities

GST activity was determined by spectrophotometry at 340 nm in 100 mM sodium phosphate buffer (pH = 6.5), 20 µM 1-chloro-2,4-dinitrobenzene, and 50 µM reduced glutathione (GSH), following the method described by Habig et al. [48] and adapted for mammal serum GST activity by Habdous et al. [49]. CbE activity was measured using 1-naphthyl acetate (1-NA) as the substrate [50]. Briefly, the hydrolysis of 1-NA was determined according to the method of Gomori [51] that was adapted by Bunyan and Jennings [52]. GST and CbE activities were expressed as nmol min^−1^ mg^−1^ of proteins.

The activities of ALP, ALT, and AST were measured using commercial kits (Wiener Lab^®^, Rosario, Argentina) according to the manufacturer’s instructions and standardized procedures [50,53]. ALP, ALT, and AST activities were expressed as U mg ^−1^ of proteins.

##### Thyroid Hormone Levels

Total T4 levels were determined using enzyme-linked electrochemical luminescent immunoassay (ECLIA) kits (COBAS^®^, Roche Diagnostics, Indianapolis, IN, USA), according to the manufacturer’s instructions. The detection limit for T4 was 0.42 ng g^−1^ [54].

#### 2.4.2. Genotoxic Biomarkers

The micronucleus (MN) test was carried out following the protocol of Cabagna et al. [55] and Lajmanovich et al. [56,57]. Briefly, blood smears were prepared on slides, fixed, and stained with May–Grünwald–Giemsa stain. Coded and randomized slides were scored by a single blinded observer. The MN frequency was determined in 1000 erythrocytes from each tadpole using a microscope under 100× magnification.

The presence of other erythrocyte nuclear abnormalities (ENAs) was also determined in mature erythrocytes according to Carrasco et al. [58], Guilherme et al. [59], and Lajmanovich et al. [57], and classified into the following types: lobed nuclei, binucleated, notched nuclei, kidney-shaped nuclei, pyknotic nuclei, and erythroplastids or anucleated erythrocytes. The results were expressed as ENA frequency and the mean value of the sum of all the lesions observed [57].

#### 2.4.3. Physiological Biomarkers

##### Heart Rate

The heart rate (HR) was evaluated following the methodology described by our group for other native amphibian species [60,61]. Briefly, tadpoles (n = 7 per treatment) were placed in ventral side up position in a thin concave plate. The heart area was bottom-up trans-illuminated with a spot-led cold light (Luxeon Rebel 3 watt LED©Philips Lumileds, San Jose, CA, USA). Videos were recorded with a remote-triggered portable USB Digital Microscope (video capture resolution: 640 × 480, 30 fps) in lab conditions (constant temperature of 24 °C) for 15 s. The HR (beats min^−1^) was quantified from slow-speed digital videos by direct visual examination of maximum systole ventricle beating [61,62]. The number of beats measured in the 15 s recorded was multiplied by four to obtain the value for 1 min [60,63].

#### Fecal Pellet Production (FPP)

FPP is an effective non-invasive measure that could be used to estimate the index of consumption, toxic effect, and damage to the gastrointestinal tract and physiological stress in amphibian tadpoles [64,65]. The FPP by individuals exposed to each treatment was measured for 48 h. Sagittal photographs of the tanks were taken for each treatment replicate before the renewal of solutions. The photographed fields were processed with Image J^®^ software version 1.54f. Threshold settings were set to enable measurement of the total bottom area (TBA) of the tank and the bottom area containing fecal pellets (AFP). FPP was expressed as the coverage of the tank bottom with fecal matter (expressed as percentage of fecal cover, %FC), and calculated as %FC = (AFP.100)/TBA.

### 2.5. Data Analysis

The cumulative mortality of each treatment of the acute assay was expressed as survival rate (% of living individuals at each exposure time; *sensu* Lajmanovich et al. [66]). The LC_50_ values and their respective 95% confidence limits were calculated at 48, 72, and 96 h, using the Trimmed Spearman–Karber method, which is considered an accurate and precise calculation method for toxicological investigations [67]. The LOEC and NOEC were derived by analyzing the survival rate, and defined as the tested concentration that showed the minimal mortality and the tested concentration that did not show mortality, respectively [68]. When the LOEC was higher than the LC_50_, it was considered not applicable [69].

The data of sublethal biomarkers are expressed as mean ± SD. The Kolmogorov–Smirnov and Levene tests were performed to verify the normality and homogeneity of the variance of the biomarker data [70]. Univariate analysis of variance (ANOVA) followed by post hoc Dunnett’s test were performed for the analyses of the data sets recorded during the first week of the chronic bioassay (Li 2.5 and Li 20 treatments), whereas the unpaired Welch t test was performed for the analyses of the data sets obtained during the second week (Li 2.5 treatment). For all statistical tests, values of *p* < 0.05 were considered significant. All these statistical analyses were performed using BioEstat software 5.0 [71] and InfoStat/P version 1.1 (Grupo InfoStat Professional, Facultad de Ciencias Agrarias, Universidad Nacional de Córdoba, Argentina).

## 3. Results

### 3.1. Acute Lethality Test

**Figure 1:**
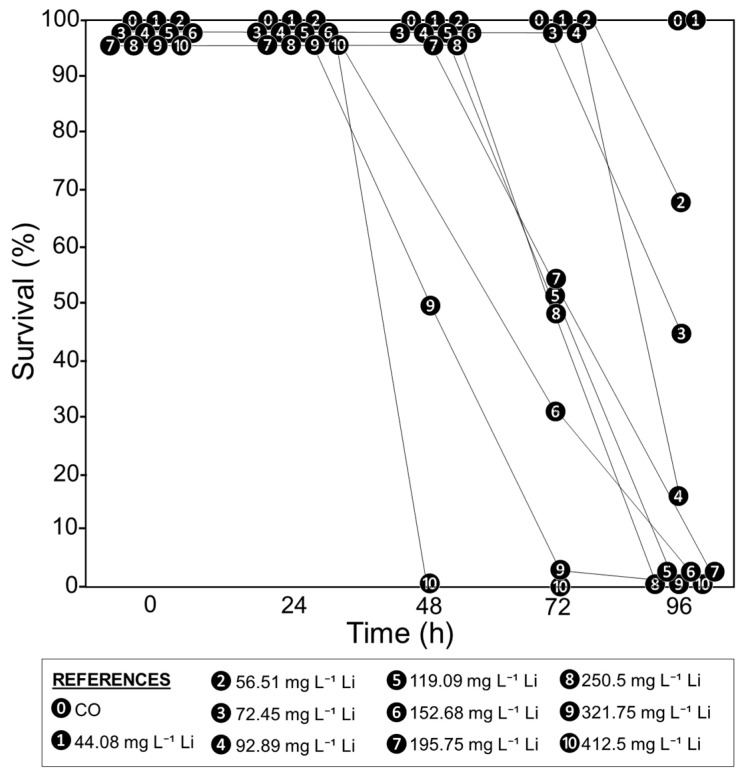
Survival rate percentages of *Rhinella arenarum* tadpoles exposed to different concentrations of Li (mg L^−1^) and a negative control (CO) at 24, 48, 72, and 96 h of exposure.

In the first 24 h, no tadpole mortality was observed in the CO and all Li treatments (survival of 100%, Figure 1). The survival rate began to decline after 48 h in the treatments with the highest Li concentrations (321.75 and 412.5 mg L^−1^). The survival rate was lower at 72 and 96 h, even in the treatments with the lowest Li concentrations (up to 56.51 mg L^−1^), as shown in Figure 1.

As the mortality in the Li treatments increased over time, the values of LC_50_, LOEC, and NOEC were lower in the longer exposure times (Table 1). The trimmed Spearman–Karber method allowed us to determine the LC_50_ values of Li for each exposure time, which ranged from 319.52 (281.21–363.05) mg L^−1^ at 48 h to 66.92 (52.76–84.89) mg L^−1^ at 96 h. In addition, the LOEC decreased from 119.09 mg L^−1^ (72 h) to 56.51 mg L^−1^ (96 h), and the NOEC from 250 mg L^−1^ (48 h) to 44.08 mg L^−1^ (96 h) (Table 1).

### 3.2. Chronic Bioassay

#### 3.2.1. Biochemical Biomarkers

**Figure 2:**
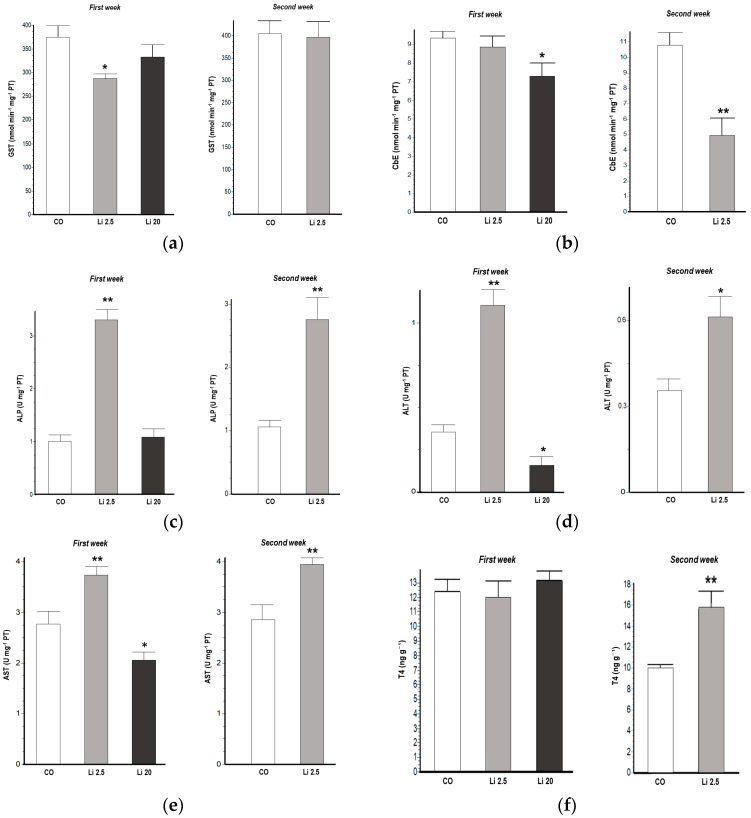
Biochemical biomarkers of *Rhinella arenarum* tadpoles exposed to negative control treatment (CO), 2.5 mg L^−1^ Li (Li 2.5), and 20 mg L^−1^ (Li 20) Li during the first week of exposure, and CO and Li 2.5 during the second week: (**a**) glutathione-S-transferase, GST; (**b**) carboxylesterase, CbE; (**c**) alkaline phosphatase, ALP; (**d**) alanine aminotransferase, ALT; (**e**) aspartate aminotransferase, AST; (**f**) thyroid hormone T4. Data are expressed as the mean ± SD. Significant differences compared to the control: * *p* ˂ 0.05; ** *p* ˂ 0.01.

GST activity significantly decreased (F = 3.61, *p* < 0.05) in the Li 2.5 treatment in the first week of exposure with respect to the CO (Figure 2a). CbE activity also significantly decreased in the first week (F = 3.55, *p* < 0.05) of exposure in the Li 20 treatment. Similarly, CbE decreased (t = 4.387, *p* < 0.05) after the second week of exposure in the Li 2.5 treatment with respect to the CO (Figure 2b).

ALP, ALT, and AST activities were significantly increased in tadpoles exposed to Li 2.5 in the first week (F = 73.58 *p* < 0.01, F = 57.22 *p* < 0.01, and F = 11.63 *p* < 0.01, respectively). Similarly, these enzyme activities were also increased in the second week of exposure (t = 4.11 *p* < 0.01, t = 2.73 *p* < 0.05, t = 3.5 *p* < 0.01, respectively). In all cases, these were increases compared to the CO (Figure 2c–e). In contrast, ALT and AST activities were significantly reduced in tadpoles exposed to Li 20 in the first week (F = 57.22 *p* < 0.05, and F = 11.63 *p* < 0.05, respectively) with respect to CO tadpoles.

The T4 levels significantly increased (t = 3.107, *p* < 0.01) in the Li 2.5 treatment in the second week of exposure with respect to the CO (Figure 2f).

#### 3.2.2. Genotoxic Biomarkers

**Figure 3:**
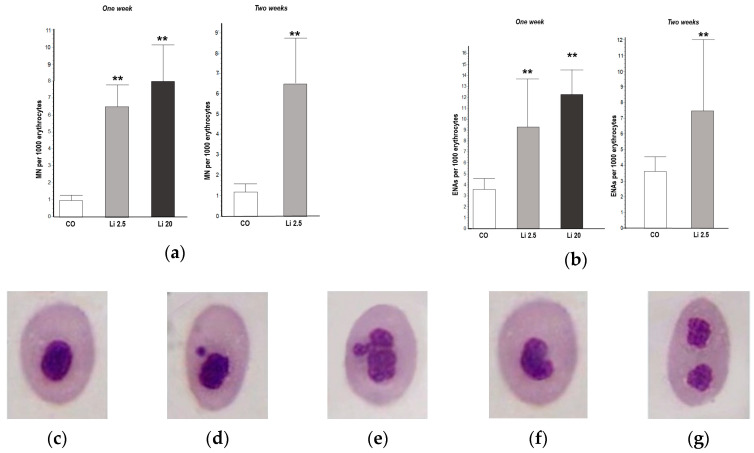
Frequencies of (**a**) micronuclei (MNs) and (**b**) other erythrocyte nuclear abnormalities (ENAs) in *Rhinella arenarum* tadpoles exposed to a negative control treatment (CO), 2.5 mg L^−1^ Li (Li 2.5), and 20 mg L^−1^ (Li 20) Li during the first week of exposure, and CO and Li 2.5 during the second week. Frequencies are expressed as the mean ± SD number of MNs or ENAs per 1000 erythrocytes. Significant differences compared to the control: ** *p* ˂ 0.01. Microphotographs (magnification 1000×) show May–Grünwald–Giemsa-stained erythrocytes: (**c**) normal; (**d**) with micronucleus; (**e**) lobulated; (**f**) with kidney-shaped nucleus; (**g**) with binucleated nucleus.

A significantly higher MN frequency was observed in the blood of all Li-treated tadpoles with respect to the CO, both in the first and second weeks of exposure (F = 29.29, *p* < 0.01, and t = 4.747, *p* < 0.01, respectively; Figure 3a). The frequency of MNs in the CO tadpoles did not exceed the mean of 1 MN per 1000 erythrocytes in the first or second week of exposure. The frequency of MNs was higher in the Li 20 treatment (8 ± 2.12 MNs per 1000 erythrocytes) in the first week than in the Li 2.5 treatment both in the first and second weeks of exposure (6.8 ± 1.48 and 6.4 ± 2.3 per 1000 erythrocytes, respectively).

The frequency of ENAs was significantly higher (F = 16.93, *p* < 0.01; Figure 3b) in both Li treatments in the first week of exposure (9.25 ± 4.35 and 12.25 ± 2.21 ENAs per 1000 erythrocytes for Li 2.5 and Li 20, respectively) with respect to the CO (3.25 ± 0.97 ENAs per 1000 erythrocytes). The frequency of ENAs was also higher (t = 2.67 *p* < 0.01) in Li 2.5 in the second week of exposure (7.4 ± 4.76 ENAs per 1000 erythrocytes) compared to the CO (3.5 ± 0.95). The most frequent types of ENAs observed in all treatments were erythrocytes with lobed and kidney-shaped nuclei, followed by those with binucleated (Figure 3e–g) and notched nuclei.

#### 3.2.3. Physiological Biomarkers

##### Heart Rate

**Figure 4:**
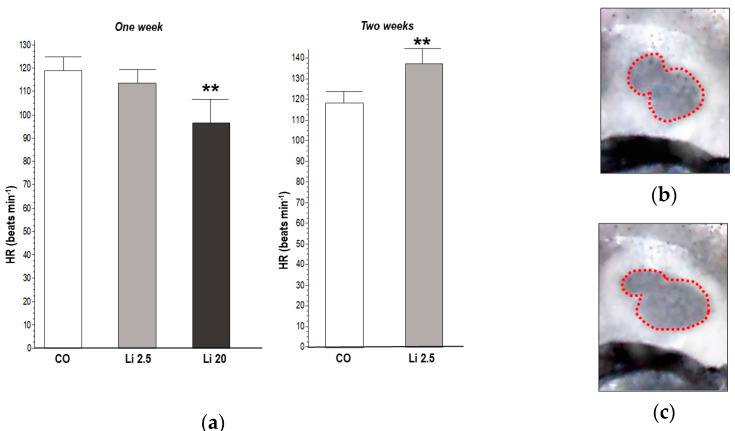
(**a**) Heart rate (HR) of tadpoles of *Rhinella arenarum* exposed to a negative control treatment (CO), 2.5 mg L^−1^ Li (Li 2.5), and 20 mg L^−1^ (Li 20) Li for one week, and CO and Li 2.5 for two weeks. Images show phases of the cardiac cycle rhythm used to measure the beats: ventricular systole (**b**) and diastole (**c**). Significant differences compared to CO ** *p* ˂ 0.01.

The HR significantly decreased (F = 7.56, *p* ˂ 0.01) in tadpoles exposed to Li 20 for one week (97.6 ± 10.8 beats min^−1^) with respect to the CO (119.6 ± 4.56 beats min^−1^). In contrast, the HR significantly increased (t = 3.55, *p* ˂ 0.01) in tadpoles exposed to Li 2.5 for two weeks (138.4 ± 11.52 beats min^−1^) with respect to the CO (118.8 ± 4.38 beats min^−1^). The results of the HR analysis are shown in Figure 4.

##### Fecal Pellet Production (FPP)

**Figure 5:**
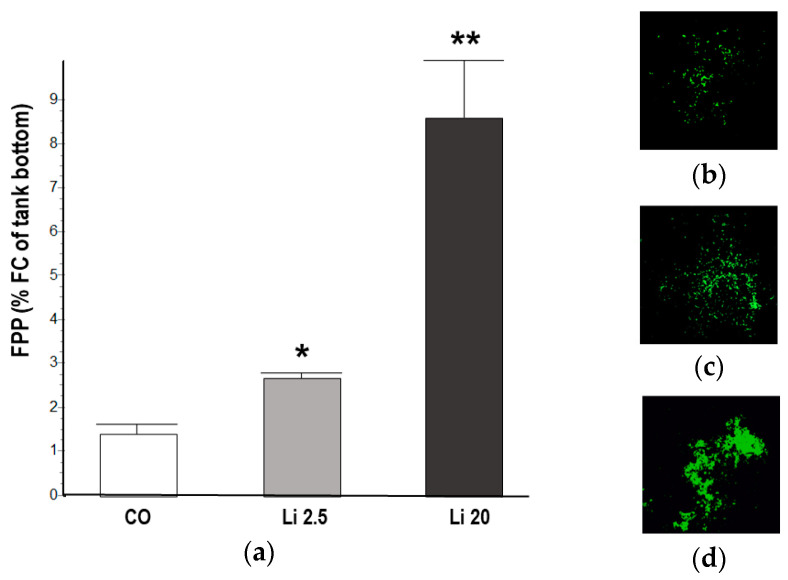
(**a**) Fecal pellet production (FPP) of *Rhinella arenarum* tadpoles exposed to a negative control treatment (CO), 2.5 mg L^−1^ Li (Li 2.5), and 20 mg L^−1^ (Li 20) for 48 h, expressed as percentage of fecal coverage (%FC) of the tank bottom. The images show the vectors obtained from the photographs of the bottom of the tanks of each treatment, (**b**) CO, (**c**) Li 2.5, and (**d**) Li 20, for the calculation of %FC. Significant differences compared to CO: * *p* ˂ 0.05; ** *p* ˂ 0.01.

The tadpoles exposed to the Li 2.5 and Li 20 treatments showed significantly higher FPP than the CO tadpoles (F = 147.13, *p* ˂ 0.01). At 48 h of exposure, the FPP in the CO was 1.41 ± 0.26% FC, whereas that in Li 2.5 was 2.69 ± 0.9% FC and that in Li 20 was 8.47 ± 1.39% FC (Figure 5).

## 4. Discussion

The ecotoxicity of Li has been determined in different aquatic organisms, including algae, cladocerans, insects, and fish [27,72,73,74]. As in our study, in these ecotoxicological studies, the form of Li tested was Li chloride (LiCl) [21,38]. It is important to point out that studies on amphibians remain scarce and that only a few have focused on the effects of Li at early embryonic stages [74]. The results of our present study highlight that, at environmentally relevant concentrations, Li is highly ecotoxic for advanced development stages of *R. arenarum* tadpoles after both a short (acute bioassay) and a medium (medium chronic bioassay, two weeks) exposure time. After a long exposure, sublethal responses can lead to irreversible effects on growth and development (during all embryogenesis and metamorphosis stages) [75,76].

In addition, the results obtained in the acute bioassay performed in the present study suggest that the effects of Li on the mortality of amphibian tadpoles are dependent on both the development stage and time of exposure. The LC_50_ at 96 h (66.92 mg L^−1^ Li) decreased by approximately 80% compared to the value obtained at 48 h (319.52 mg L^−1^ Li). In amphibians, a much lower value of the 96 h LC_50_ of 2 mg L^−1^ has been reported for *Xenopus laevis* embryos [19]. The fact that acute mortality was significantly higher at high Li concentrations after a short exposure can be explained in terms of the sensitivity of the development stages evaluated (the susceptibility to the contaminant depends on the period of development during which the exposure occurs, with embryos being much more sensitive than tadpoles) [19]. The wide range of LC_50_ values obtained in this study according to the day of exposure is consistent with other studies that reported varied 96 h LC_50_ values in several fish species, ranging from 13 to more than 100 mg L^−1^ Li at 96 h, in contrast with a lower range with longer exposures, reaching values of 0.6 and 1.4 mg L^−1^ at 26 days [73,77]. This belated effect of Li also probably influenced the unexpected mortality of tadpoles in the Li 20 treatment in the chronic bioassay after the first week. Although Li 20 is far below the NOEC value in the acute bioassay, a longer exposure could also mean lethal effects. In an acute exposure, Li may not yet completely be absorbed into the body, and therefore not all its effects would be displayed [74,78]. Furthermore, the fact that a concentration estimated to be non-lethal has shown mortality could also explain the antagonistic or hormetic effect observed for certain biomarkers such as GST, ALT, ALP, and AST relative to the lowest non-lethal treatment (Li 2.5). These differences could be due to a metabolic depletion that organisms exposed to Li 20 may be undergoing through the exposure before death, which leads to a drastic decrease in the normal functioning of their metabolism.

Since enzymes such as GST and CbE play a key role in the detoxification of xenobiotics and their metabolism, they are expected to be increased under toxic exposure scenarios [79,80]. However, in *R. arenarum* tadpoles exposed to Li, the activities of both enzymes were inhibited. Mainly, GST activity was significantly decreased in the Li 2.5 treatment group after the first week of exposure, a fact that may be explained in terms of its inactivation by Li or due to the depletion of glutathione conjugate, which leads to the loss of GST activity [81]. As GST contributes to protecting tissues from oxidative stress, its decrease could suggest alterations in the antioxidant defense system [60]. In addition, the higher sensitivity of GST at the lowest concentration and exposure time could also suggest a physiological adaptation to Li, as was already described for tadpoles of *R. arenarum* exposed to other pollutants such as pesticides and antibiotics [44].

Regarding CbE, this enzyme plays an important role in xenobiotic metabolism. Similarly, to our results, some other studies have reported CbE inhibition by different compounds (agrochemicals and microplastics) in amphibian tadpoles [82,83,84]. In our study, a decrease in CbE activity was observed in tadpoles treated with Li 20 for one week as well as in tadpoles treated with Li 2.5 for two weeks, suggesting a medium-long term inhibitory effect of Li on this enzyme. This result is not consistent with previous studies on Li effects on CbE, since varied responses of the enzyme have been reported in different animals. For instance, Prabha et al. [85] found that Li has a positive effect on the release of CbE in rats, whereas Cunha et al. [86] showed no significant effects of Li exposure on CbE in the mussel *Mytilus galloprovincialis*. More studies with longer exposure times are needed to better understand the effects of Li on these enzymes and the biological processes in which they are involved, as well as the mechanisms by which the response occurs.

In addition to the alterations in the detoxification process, observed by the modification of the activity of key enzymes such as GST and CbE, the tadpoles exposed to Li also showed alterations in ALT, AST, and ALP activities, suggesting alterations in amino acid metabolism and tissue damage. In particular, an increase in AST and ALT indicates damage to hepatic tissues [87]. Also, an increase in ALP indicates an obstruction in the intra- and extrahepatic biliary system [53]. A more colored gallbladder compared to control tadpoles was also observed in larvae exposed to Li (pers. obs.), which may support this hypothesis. ALP is a key enzyme in many biological processes related to the detoxification, biosynthesis, and metabolism of energetic macromolecules, and alterations in its activities are linked to alterations in the permeability of the plasma membrane or cellular necrosis [88]. Consistent with this, several cytotoxic effects of Li exposure, like necrosis, vacuolization, and inflammation of several tissues, have been reported [89,90]. More studies should be performed to elucidate the degree of the cytotoxic effects and inflammation of tissues.

The activities of ALT and AST showed the same pattern of increase in Li 2.5-treated tadpoles and decrease in Li 20-treated tadpoles. These results highlight a Li concentration-dependent response of these enzymes. A comparative decrease in the activity of both enzymes at higher concentrations has also been recorded in rats exposed to LiCl [91]. Previous studies also suggest a time-dependent response of both ALT and AST due to the normal activity found in acute Li exposure in contrast to their increased activity after one week of Li exposure [92].

Regarding the thyroid hormone T4, its levels increased after two weeks of exposure to Li 2.5. This result suggests an effect of long-term Li exposure on this hormone, since no significant differences in T4 levels were observed for the same treatment with respect to the CO after the first week of exposure. Pinto et al. [38] also suggested an increase in T4 availability in tadpoles of the American bullfrog (*Lithobates catesbeianus*) exposed to 2.5 mg L^−1^ Li. Moreover, an overproduction of T4 has also been reported by Palmér et al. [93] as an effect of Li toxicity in humans. Similar findings have been reported by Benzd et al. [94], who also proposed an association between the duration of the exposure and the degree of persistence of the effect. In addition, T4 levels could be related to the inactivation of the sensing receptors of Ca^2+^ by Li, and interference with signaling that may enhance thyroid production and Ca^2+^ in blood [95]. In accordance with the similarity of T4 and its mechanisms of action in vertebrates, the endocrine disruption mechanism of Li suggested for humans could also explain the Li thyrotoxicosis observed in amphibian tadpoles [96]. In addition, the increase in T4 in amphibians could also be caused by corticosterone, as, in many cases of stress due to contaminants, it leads to an acceleration of metamorphosis to allow the animal to leave the contaminated environment [38,97].

Regarding genotoxic biomarkers, the high frequency of MNs and other ENAs observed in Li-treated tadpoles indicates a strong genotoxic effect. Some authors [57,72,73] have proposed that variations in the shape of red blood cells and a high frequency of ENAs could indicate a good complementary approach for detecting genotoxicity damage. The increased frequency of these nuclear abnormalities is indicative of an adverse cellular reaction and/or a surveillance mechanism to eliminate cells with genetic damage [57]. The occurrence of erythrocytes with lobulated nuclei and segmented cytoplasm is considered indicative of direct cell division (amitosis), which, along with mitotic cell proliferation, could represent a short-term means for increasing the oxygen-carrying capability of the blood in amphibian tadpoles [77]. The genotoxicity of Li has been widely studied in model organisms such as mice through the macronucleus test and study of chromosome aberrations [98]. The effects of high concentrations of Li exposure include conformational changes in DNA as well as the inhibition of protein and amide biosynthesis [99]. It has been suggested that DNA damage could be due to the oxidative stress induced by Li [29,100]. The mechanisms involved in Li-induced DNA damage have been deeply studied and are mostly related to Li-induced downregulation of p57, a multifunctional protein related to the control of gene expression, growth, differentiation, cytoskeletal organization, and apoptosis [101,102].

Finally, regarding physiological markers, tadpoles exposed to Li 20 for one week showed a lower heart rate (HR) than the CO tadpoles, while tadpoles exposed to Li 2.5 for two weeks showed a higher HR than the CO tadpoles. Alterations in the heart beat rhythm could be associated with Li interference with the cellular ionic homeostasis of Na+ and K+, altering the voltage of the myocytes and cause electrical instability in the heart ventricle [103,104]. Since Li is used in pharmacology and medicine as part of psychiatric treatments, its side effects on key organs such as the heart are well studied in humans [105]. The cardiotoxic effects of Li, including sinoatrial block, intraventricular conduction delay, arrhythmias, and prolongation or shortening of different parts of the cardiac cycle, have already been reported in humans [28]. In addition, Truedson et al. [78] found that Li can affect the heart beat rate at different exposure times, including chronic, acute and even therapeutic conditions. It is more than clear that Li has a cardiotoxic effect not only in humans, but also in non-target organisms such as amphibians, as demonstrated in our study. More studies are necessary to elucidate the mechanisms involved in these cardiac alterations due to Li in wildlife and its consequences on the survival of organisms.

Lastly, the increased FPP observed in tadpoles exposed to Li for 48 h suggests a clear sign of diarrhea. Gastrointestinal alterations including diarrhea are one of the most commonly described acute effects of Li [35,106]. Moreover, Lei et al. [107] reported that LiCl exposure causes colonic inflammation and gut microbiota dysbiosis as well as the downregulation of IgA, which is related to intestinal immunity in mice. To our knowledge, this is the first study that uses FPP as a physiological biomarker in amphibians. The use of FPP proved to be useful since it allowed us to obtain quick, inexpensive, and non-invasive results. In addition, it can be used as a standard method for future studies with classical biomarkers to explore the loss of food energy storage and the effects of Li on gastrointestinal function, such as effects on histological and bacterial microbiota [44],.

## 5. Conclusions

The results of our study show that exposure to environmentally relevant concentrations of Li causes high ecotoxicity in amphibian tadpoles. The biomarker responses demonstrated genotoxicity, thyrotoxicosis, cardiotoxicity, loss of food energy storage via gastrointestinal dysfunction, and a decrease in the enzymatic profiles of antioxidant and biotransformation systems, which lead to an imbalance between the generation and elimination of reactive oxygen species. These alterations provide the first evidence of Li ecotoxicity in native species of Argentina and serve as an early warning for organisms whose distribution includes the Li Triangle. In addition, it is important to point out that, even when exposed to low concentrations that are considered environmentally safe (2.5 mg L^−1^ Li), *R. arenarum* tadpoles showed severe responses, which may represent an ecotoxicological concern. Even more worrying, tadpoles exposed to the highest concentration in the chronic bioassay, which represents environmental Li concentrations (20 mg L^−1^ Li) in wetlands and freshwater near mining areas, showed significant mortality after the first week of exposure.

These environmental risk evaluations and biomarker responses in amphibian tadpoles are far from what long-term sustainability and protection of biodiversity means in the annual reports of multinational Li companies that are exploiting the Li Triangle in Argentina. It is important to point out that, although the Escazú Protocol is approved in Argentina and considering that ecological health is an issue that concerns everything that is within the environment (eco-centric vision), public and scientific contributions to environmental decisions and management is not allowed by current governments. This new “green” energy revolution, which has created a high global demand for Li, a contaminant of emerging concern in aquatic systems, is likely a future “timebomb”.

## Figures and Tables

**Table 1:** Values of the median lethal concentration (LC_50_) and the no- and lowest-observed-effect concentrations (NOEC and LOEC, respectively) of Li (mg L^−1^) for *Rhinella arenarum* tadpoles (GS 26).

	48 h	72 h	96 h
LC_50_	319.52 (281.21–363.05) mg L^−1^	167.026 (107.68–246.47) mg L^−1^	66.92 (52.76–84.89) mg L^−1^
LOEC	n.a.	119.09 mg L^−1^	56.51 mg L^−1^
NOEC	250 mg L^−1^	92.98 mg L^−1^	44.08 mg L^−1^

n.a.: not applicable since the LOEC value was higher than the LC_50_.

## Data Availability

The data presented in this study are available on request from the corresponding authors.
